# P-376. The US REGAL Cohort: A Retrospective Real-world Study of the Effectiveness and Tolerability of the Antiretroviral Treatment Regimens DTG/3TC Compared to BIC/FTC/TAF in Older Persons Living with HIV

**DOI:** 10.1093/ofid/ofaf695.594

**Published:** 2026-01-11

**Authors:** Onyema Ogbuagu, Jeremy Fraysse, Jennifer Kuretski, Gustavo Verdier, Cynthia Firnhaber, Emilio Letang, Tanya Schreibman, Cassidy Henegar, Rebecca Glassman, Deanna Merrill, Carly Rodriguez, Richard Grove, Paula Peressini López, Bryn Jones, Julie Priest

**Affiliations:** Yale School of Medicine, New Haven, CT, USA, New Haven, Connecticut; ViiV Healthcare, Durham, NC; Midway Specialty Care Center, West Palm Beach, Florida; ViiV Healthcare, Montréal, QC, Canada, Pointe-Claire, Quebec, Canada; University of Colorado Anschutz Medical Center, Denvier, Colorado; ViiV Healthcare, Madrid, Spain, Madrid, Madrid, Spain; CAN Community Health, Sarasota, Florida; ViiV Healthcare, Durham, NC; Westchester Medical Center Health Network, Hawthorne, New York; ViiV Healthcare, Durham, NC; IQVIA, Durham, North Carolina; GlaxoSmithKline, Brentford, UK, Brentford, England, United Kingdom; IQVIA, Durham, North Carolina; ViiV Healthcare, Durham, NC; ViiV Healthcare, Durham, NC

## Abstract

**Background:**

Older persons living with human immunodeficiency virus (PLHIV) have more age-related comorbidities than the general population and greater potential for polypharmacy and drug-drug interactions with their antiretroviral treatment (ART). Data comparing the real-world effectiveness of the two-drug dolutegravir/lamivudine (DTG/3TC) and three-drug bictegravir/emtricitabine/tenofovir alafenamide (BIC/FTC/TAF) regimens is limited in older PLHIV.

**Figure:**
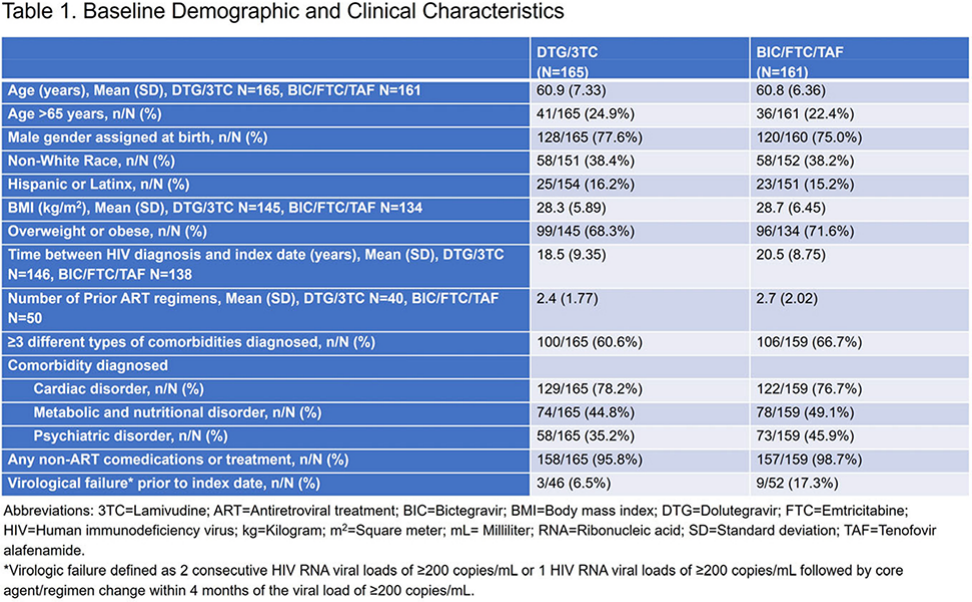

**Figure:**
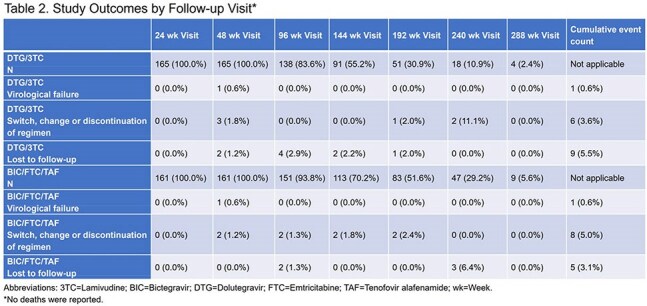

**Methods:**

To assess the real-world effectiveness of switching to DTG/3TC versus BIC/FTC/TAF in older PLHIV, we conducted a retrospective chart review among people aged ≥50 years on ART who were virologically suppressed with ≥24 weeks of follow-up in the United States (US). Index date was defined as DTG/3TC or BIC/FTC/TAF initiation date.

**Results:**

Among 326 PLHIV across 5 study sites, 165 were on DTG/3TC and 161 were on BIC/FTC/TAF. PLHIV were on average aged 61 years (23.6% aged >65 years), 76.3% male gender, 38.3% non-White, 15.7% Hispanic/Latinx ethnicity, and diagnosed with HIV an average of 19 years before index date. Clinical characteristics were similar between groups at index date (Table 1). A high burden of age-related comedications and comorbidities was observed. A total of 97.2% of PLHIV reported non-ART comedications. Approximately 64% of PLHIV reported ≥3 comorbidities; cardiac disorders (77.5%), metabolic and nutritional disorders (46.9%), and psychiatric disorders (40.4%) were most common. Prior virological failure was reported in 6.5% of PLHIV on DTG/3TC and 17.3% of PLHIV on BIC/FTC/TAF. Total follow-up was 356.6 and 435.8 person-years in DTG/3TC and BIC/FTC/TAF groups, respectively. Approximately 20% of PLHIV had 240 weeks of follow-up. Virological failure was reported for 1 PLHIV in each group. The incidence rate of virological failure was 0.28 (95% confidence interval [CI]: 0.01, 0.59) and 0.23 (95% CI: 0.01, 0.48) per 100 person-years for DTG/3TC and BIC/FTC/TAF, respectively over the study period. No treatment-emergent resistance was reported.

**Conclusion:**

In older, virologically suppressed PLHIV with significant burden of age-related comorbidities and comedications in the US, switching to either two-drug DTG/3TC or three-drug BIC/FTC/TAF maintained long-term viral suppression without resistance.

**Disclosures:**

Onyema Ogbuagu, MA, FACP, FIDSA, Gilead Sciences, Inc.: Advisor/Consultant|ViiV: Advisor/Consultant Jeremy Fraysse, MS, GSK: Stocks/Bonds (Public Company)|ViiV Healthcare: Stocks/Bonds (Private Company) Jennifer Kuretski, DNP, APRN, NP-C, AAHIVS, Gilead Sciences: Speaker|ViiV: Speaker Gustavo Verdier, BSc, BPharm, MBA, ViiV Healthcare: Employee Cynthia Firnhaber, MD, MS, DTM&H, MERCK: Advisor/Consultant|MERCK: Grant/Research Support Emilio Letang, MD, MPH, PhD, GSK: Stocks/Bonds (Public Company)|ViiV Healthcare: Employee Cassidy Henegar, PhD, MSPH, ViiV Healthcare: Employee|ViiV Healthcare: Stocks/Bonds (Public Company) Rebecca Glassman, MD, Gilead: Advisor/Consultant Deanna Merrill, PharmD, MBA, AAHIVP, ViiV Healthcare: employee Carly Rodriguez, PhD, Viiv Healthcare: Services via contract research organization Richard Grove, MSc, GSK: Stocks/Bonds (Public Company) Paula Peressini López, PhD, ViiV Healthcare: Financial payments to conduct the study Bryn Jones, MBChB, GSK: Stocks/Bonds (Public Company)|ViiV Healthcare: Employee Julie Priest, MSPH, GSK: Stocks/Bonds (Public Company)|ViiV Healthcare: Employee

